# Improving ECG Classification Accuracy Using an Ensemble of Neural Network Modules

**DOI:** 10.1371/journal.pone.0024386

**Published:** 2011-10-26

**Authors:** Mehrdad Javadi, Reza Ebrahimpour, Atena Sajedin, Soheil Faridi, Shokoufeh Zakernejad

**Affiliations:** 1 Islamic Azad University, South Tehran Branch, Tehran, Iran; 2 Brain and Intelligent Systems Research Lab, Department of Electrical and Computer Engineering, Shahid Rajaee Teacher Training University, Tehran, Iran; 3 School of Cognitive Sciences (SCS), Institute for Research in Fundamental Sciences (IPM), Tehran, Iran; Rutgers University, United States of America

## Abstract

This paper illustrates the use of a combined neural network model based on Stacked Generalization method for classification of electrocardiogram (ECG) beats. In conventional Stacked Generalization method, the combiner learns to map the base classifiers' outputs to the target data. We claim adding the input pattern to the base classifiers' outputs helps the combiner to obtain knowledge about the input space and as the result, performs better on the same task. Experimental results support our claim that the additional knowledge according to the input space, improves the performance of the proposed method which is called Modified Stacked Generalization. In particular, for classification of 14966 ECG beats that were not previously seen during training phase, the Modified Stacked Generalization method reduced the error rate for 12.41% in comparison with the best of ten popular classifier fusion methods including Max, Min, Average, Product, Majority Voting, Borda Count, Decision Templates, Weighted Averaging based on Particle Swarm Optimization and Stacked Generalization.

## Introduction

Accurate and computationally efficient means of classifying electrocardiography (ECG) arrhythmias has been the subject of considerable research effort in recent years. Electrocardiography deals with the electrical activity of the heart [1]. Monitored by placing sensors at the limb extremities of the subject, electrocardiogram (ECG) is a record of the origin and the propagation of the electrical potential through cardiac muscles. It provides valuable information about the functional aspects of the heart and cardiovascular system. Early detection of heart diseases/abnormalities can prolong life and enhance the quality of living through appropriate treatment. Therefore, numerous research works analyzing the ECG signals have been reported [2]–[4]. For effective diagnostics, the study of ECG pattern and heart rate variability signal may have to be carried out over several hours. Thus, the volume of the data becomes enormous which then results in a tedious and time consuming study. Naturally, the possibility for the analyst to miss (or misread) vital information is high. Therefore, computer-based analysis and classification of diseases can be very helpful in diagnostics [5]–[10].

Several algorithms have been developed for the detection and classification of the ECG signals [11]–[14]. ECG features can be extracted in time domain [15]–[18], in frequency domain [18]–[19], or represented as statistical measures [12]. The results of the studies have demonstrated that the Wavelet Transformation is the most promising method to extract features from the ECG signals [2] [5]–[6] [10]. Wavelet Transformation opens a category of methods that represent the signal in different translations and scales. Moreover, the Discrete Wavelet Transformation decomposes a signal into different coarse signals. Wavelet coefficients obtained from the decomposition process are considered as the filtered signal in the sub bands. Features extracted from these coefficients can efficiently represent the characteristics of the original signal in different details [20]–[21]. Researchers have also demonstrated that the feature extraction methods such as Fourier Transform [22], Principal Component Analysis [23] and Independent Component Analysis [24] can be successfully employed to extract appropriate features for classification tasks.

As for classifiers, Artificial Neural Networks have been used in a great number of medical diagnostic decision support system functions obtained after dilatation and translation of an analyzing wavelet [25]–[27]. Among them, the Multi Layer Perceptrons (MLPs) [16]–[19] and Radial Basis Function [3] [28]– neural networks are probably the most popular. Combining classifiers to achieve higher accuracy is an active field of research with application in the area of ECG beat classification. Essentially, the idea behind combining classifiers is based on the so-called divide-and-conquer principle, according to which a complex computational task is solved by dividing it into a number of computationally simple tasks and then combining the solutions of those tasks [30]–[32]. For example Übeyli [33] has demonstrated that the combined eigenvector methods (RNN approach) can be useful in analyzing the ECG beats. Osowski et al. [34] have used an ensemble of neural networks for recognition and classification of arrhythmia. The implementation of Multiclass Support Vector Machine with the Error Correcting Output Codes is presented for classification of electrocardiogram (ECG) beats in ref [35]. There are two main strategies in combining classifiers: fusion and selection [36]. In classifier fusion, it is supposed that each ensemble member is trained on the whole problem space [37], whereas in classifier selection, each member is assigned to learn a part of the problem space [38]–[40]. This way, in the former strategy, the final decision is made considering the decisions of all members, while in the latter strategy, the final decision is made by aggregating the decisions of one or a few of experts [41]–[42]. Combining classifiers based on the fusion of outputs of a set of different classifiers has been developed as a method for improving the recognition rate of classification problems [43]–[45]. The general framework using an ensemble of neural classifiers in two levels is often referred to as Stacked Generalization [46]. In the first level, various neural classifiers are used to learn different models from the original dataset. The decisions of the first level classifiers and the corresponding target class of the original input data are then used as the input and target to learn the second level classifier, respectively.

In this paper, we propose a new combination method for classifying normal heartbeats, Premature Ventricular Contraction (PVC) and other abnormalities. In the preprocessing module, an Undecimated Wavelet Transform is used to provide an informative representation that is both robust to noise and tuned to the morphological characteristics of the waveform features. For feature extraction, we have used a suitable set of features that consists of both morphological and temporal features. This way we can keep both spatial and temporal information of signals. For classification we have used a number of diverse MLPs neural networks as the base classifiers that are trained by Back Propagation algorithm. Then we employed and compared different combination methods. In our proposed method, unlike the Stacked Generalization, the second level classifier (combiner) receives the input pattern directly adding on the base classifiers outputs. In fact, in the learning phase, the combiner learns the expertise areas for each base classifier. In the test phase, based on spatial position of the input data, and by considering the expertise areas of all base classifiers, the combiner specifies the weights for optimal combination of the decisions from the base classifiers.

Therefore, we expect that the Modified Stacked Generalization method to be able to use both fusion and selection mechanisms for various test samples, proportional to the position of the sample in the problem space. We used 10 different combination methods: Max, Min, Average, Product, Majority Voting, Borda Count, Decision Templates, Weighted Averaging based on Particle Swarm Optimization, Stacked Generalization and Modified Stacked Generalization. Experimental results indicate that our proposed combining method performs better than other combining methods.

## Materials and Methods

### Data preparation

**Figure 1:**
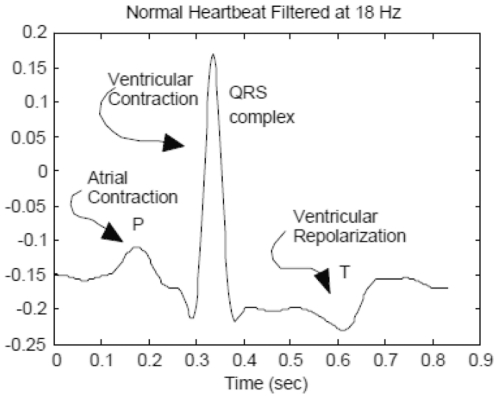
waveform of ECG signal: normal beat.

**Figure 2:**
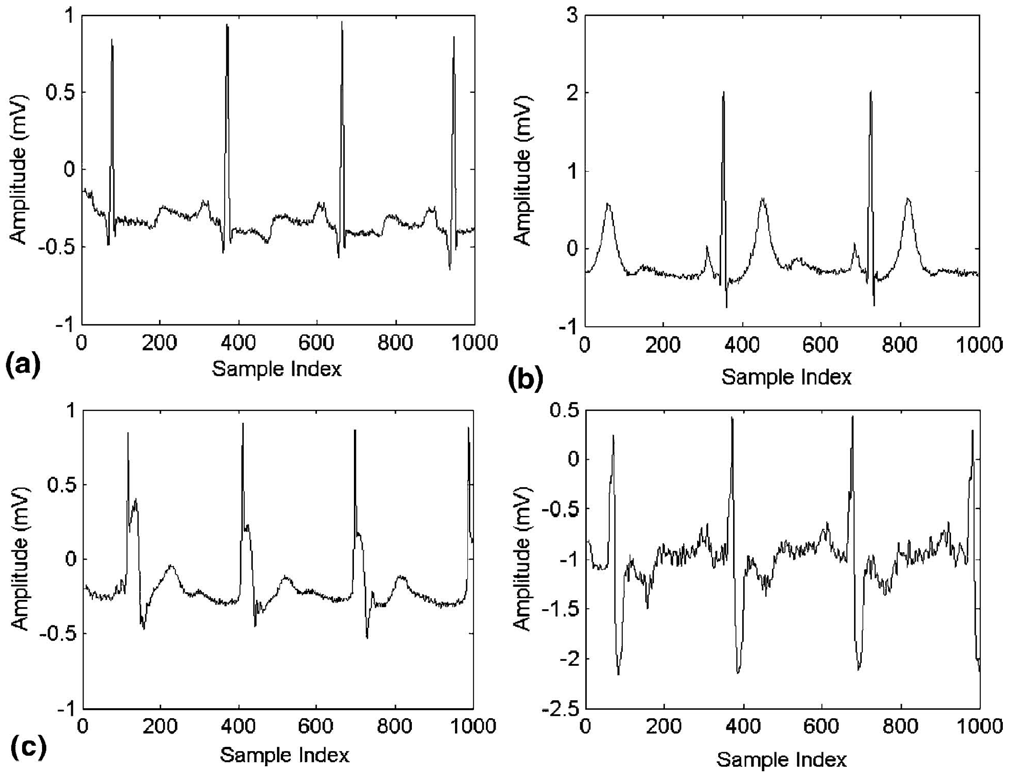
ECG signals: (a) Normal Sinus rhythm beats; (b) Premature Venticular contraction beats; (c) other beats (non conducted P-wave and right bundle branch block beats respectively).

An ECG consists of three basic waves: the P, QRS, and T. These waves correspond to the far field induced by specific electrical phenomena on the cardiac surface, namely, a trial depolarization (P wave), ventricular depolarization (QRS complex), and ventricular repolarization (T wave). One of the most important ECG components is the QRS complex [12]. Figure 1 shows a waveform of normal signal. Among the various abnormalities related to functioning of the human heart, premature ventricular contraction (PVC) is one the most important arrhythmias. PVC is the contraction of the lower chambers of the heart (the ventricles) that occur earlier than usual, because of abnormal electrical activity of the ventricles. PVC is related to premature heart beats that provide shorter RR intervals than other types of ECG signals. Changes in the RR intervals play an important role in characterizing these types of arrhythmias. Hence, we exploit the instantaneous RR interval as another feature component, which is defined as the time elapse between the current and previous R peaks [15]–[17]. This paper investigates the detection and classification of PVC arrhythmias. In Figure 2, ECG signals of three classes are shown.

The MIT–BIH arrhythmia database [47] was used as the data source in this study. The database contains 48 recordings each of which has a duration of 30 minutes and includes two leads; the modified limb lead II and one of the modified leads V1, V2, V4 or V5. The sampling frequency is 360 Hz; the data are band-pass filtered at 0.1–100 Hz and the resolution is 200 samples per mV. Twenty-three of the recordings are intended to serve as a representative sample of routine clinical recordings and 25 recordings contain complex ventricular, junctional and supra ventricular arrhythmias.

There are over 109,000 labeled ventricular beats from 15 different heartbeat types. There is a large difference in the number of examples in each heart beat type. The largest class is “Normal beat” with about 75,000 examples and the smallest class is “Supra ventricular premature beat” with just two examples. The database is indexed both in timing information and beat classification. We used a total of seven records marked as: 100, 101, 102, 104, 105, 106, and 107 in the database. We extracted a total of 15,566 beats: 8390 normal beats, 627 abnormal PVC arrhythmia beats, and 6549 other arrhythmia beats. We used the database index files from database to locate beats in ECG signals. Of all these 15566 beats, we used 450 beats for training, 150 beats for validation and the other 14966 for testing our networks. This way we assigned equal number of samples to each class in our training and validation phases (150 for training and 50 for validation for each class).

The objectives of preprocessing stage are the omission of high-frequency noise and the enhancement of signal quality to obtain appropriate features. ECG signal is measured on static conditions since various types of noise including muscle artifacts and electrode moving artifacts are coupled in dynamic environment. To remove such noises an advanced signal processing method, such as discrete wavelet transform denoising technique [20] should be used. This method has been emerged over recent years as a powerful time–frequency analysis and signal coding tool favored for the interrogation of complex signals. However, Discrete Wavelet Transformation is not a time-invariant transform. To solve this problem, we used the Stationary Wavelet Transform which is also known as the Undecimated Wavelet Transform or translation-invariant wavelet transform. Undecimated Wavelet Transform uses the average of several denoised signals that are obtained from the coefficients of ε-decimated Discrete Wavelet Transformation [20].

**Figure 3:**
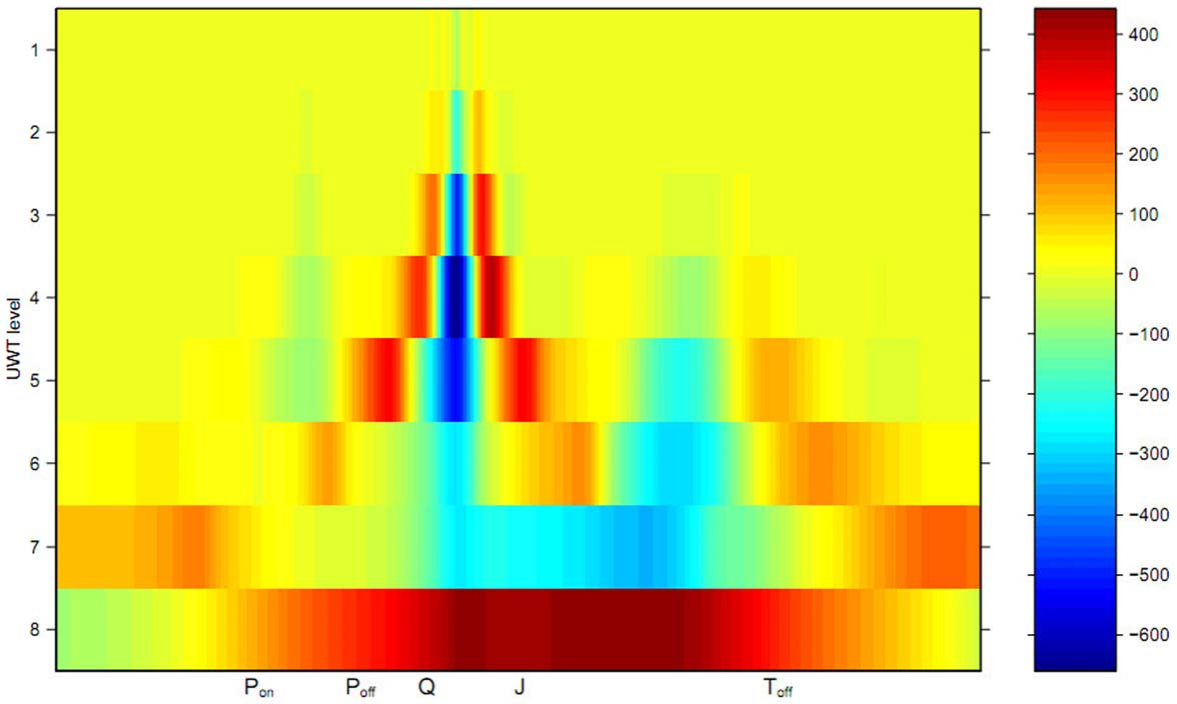
A visualization of the Undecimated Wavelet Transform coefficients for a typical ECG beat.

Figure 3 overleaf shows a color-coded visualization of the Undecimated Wavelet Transform coefficients for an ECG beat. We can see that the Undecimated Wavelet Transform coefficients can capture the joint time-frequency characteristics of the ECG waveform, particularly the QRS complex.

**Figure 4:**
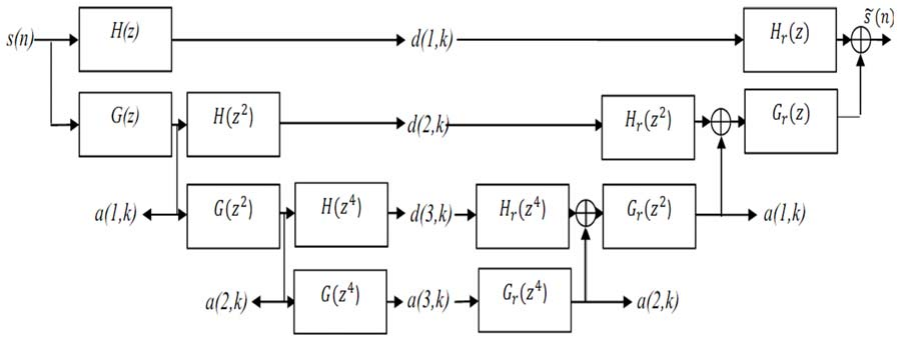
Block diagram of Undecimated Wavelet Transform. H(z) and Hr(z) are the decomposition and reconstruction high. pass filters. G(z) and Gr(z) are low pass filters. Term d(.,.) denotes the decomposition coefficients and a(., .) denotes the approximation coefficients.

Suppose the signal 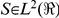. The Undecimated Wavelet Transform is given by:
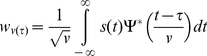
(1)where 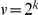, 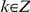, 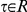 and 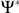 is the complex conjugate of the mother wavelet. Figure 4 shows the block diagram of Undecimated Wavelet Transform.

This figure shows a decomposition of three levels: the blocks of 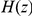 and 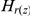 are the decomposition and reconstruction high pass filters and the blocks of 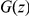 and 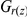 are low pass filters. 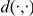 denotes the decomposition coefficients and 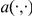 denotes the approximation coefficients. Selection of the most suitable mother wavelet filter is of great importance in biomedical signal processing in wavelet domain [48]. Although the computational load for implementing the Daubechies algorithm is higher than the other wavelet algorithms, it picks up detail that is missed by the other wavelet algorithms [49]. Even if a signal is not well represented by one member of the Daubechies family, it may still be efficiently represented by another. Selecting a wavelet function which closely matches the signal to be processed is of utmost importance in wavelet applications [50]. For example Rafiee et.al have shown that db44 is the most similar function for Electromyographic, Electroencephalographic and Vaginal Pulse Amplitude biomedical signals [48]. Daubechies wavelet family are similar in shape to QRS complex and their energy spectra are concentrated around low frequencies.

### Classification

#### Base Classifiers: Multilayer Perceptrons Neural Network

A MLPs is a supervised, fully-connected feedforward artificial neural network which learns a mapping between a set of input samples and their corresponding target classes. The MLPs is in fact an extension of the Perceptron neural network which was originally proposed by Rosenblatt in 1957 [51]. The main difference between MLPs and Perceptron is that MLPs can learn nonlinear mappings which was the paramount drawback of the Perceptron. Each node in a MLPs neural network represents a neuron which is usually considered as a nonlinear processing element. The two most popular functions to model this nonlinear behavior are 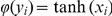 and 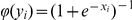 in which the former function is a hyperbolic tangent which ranges from −1 to 1, and the latter is similar in shape but ranges from 0 to 1. Here 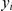 is the output of the 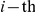 node (neuron) and 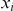 is the weighted sum of the input synapses.

A MLPs is consisted of one input layer, one or more hidden layers and one output layer. For the 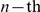 input sample, the net output of the 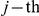 neuron in the 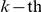 hidden layer is computed using a weighted summation over the neurons of its input:
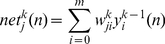
(2)where 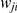 is the weight between the 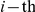 input neuron and the 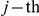 output neuron. For the first hidden layer where 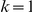, the summation is performed over the elements of the network's input which means 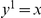. The output of each neuron 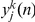 in the 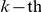 hidden layer is specified using the activation function 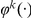
which is usually sigmoid or hyperbolic tangent. It is to be noted that the activation functions for different layers are not necessarily the same.
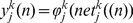
(3)The final outputs of the neural network are the values in the output layer. We try to find the optimal weights of the network during the learning process. There are various methods in the literature to train a MLPs neural network among which Back Propagation is the most popular. The general procedure to train the network starts by feeding the training samples to the network. As the initial weights of the network are determined randomly, they cannot produce the desired outputs. The goal of the learning process is to minimize the error which is defined as the difference between the outputs of the network and the desired outputs (the target classes of the input data). In order to minimize this error, we first compute 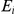 which is the error at the 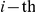 output node over the set of training instances.
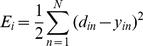
(4)where 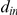 and 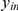 are the desired and real outputs of the 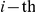 output neuron for the 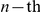 training sample and 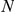 is the total number of training samples. We try to minimize the error using the Gradient Descent method in which the change for weights is:
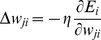
(5)where 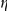 is the learning rate and is carefully selected to ensure that the weights converge to a response fast enough and without producing oscillations. It can be shown that for the sigmoid activation function the above equation results in:
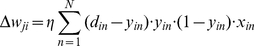
(6)


#### Combining Methodology

Combining is an approach to improve the performance in classification particularly for difficult problems such as those involving a considerable amount of noise, limited number of patterns, high dimensional feature sets, and highly overlapped classes. From a computational viewpoint, according to the principle of divide-and-conquer, a complex computational task is solved by dividing it into a number of computationally simple tasks and then combining the solutions of those tasks. In supervised learning, computational simplicity is achieved by distributing the learning task among a number of experts, which in turn divides the input space into a set of subspaces [41]. There are generally two types of combining strategies: selection and fusion [36]. The selection paradigm is based on the assumption that each of the base experts is specialized in a particular local area of the problem space. There can be one specific expert nominated to make the decision in each subspace, as was done by Rastrigin and Erenstein [39], or in some cases one can devote more than one local expert to a local area, as was done by Jacobs, Jordan, Nowlan, and Hinton [38] as well as Alpaydin and Jordan [40]. Expert fusion assumes that all experts are trained over the whole problem space, and are therefore considered as competitive rather than complementary [37] [52]. As the input signal is involved in the combining procedure, combining neural networks as experts may be classified into two major categories:

- Static structures: In this class of combining methods of neural networks, the responses of several predictors (neural networks) are combined by means of a mechanism which does not involve the input signal; hence the designation “static”.
- Dynamic structures: In the second class of combining methods, the input signal is directly involved in actuating the mechanism that integrates the outputs of the individual experts into an overall output; hence the designation “dynamic” [41].

The combination methodologies from the combiner viewpoint are divided into two categories: non-trainable and trainable. Simple algebraic combiners are, in general, non-trainable combiners of continuous outputs. In non-trainable classifiers, the total support for each class is obtained as a simple function of the supports received from individual classifiers. Following the same notation in [53], we represent the total support received by class 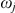, the 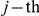 column of the decision profile 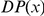, as
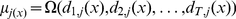
(7)where 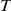 is the number of base classifiers and 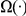 is the combination function, such as one of those listed below.

- **Mean Rule (Averaging).** The support for 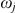, is obtained as the average of all classifiers' 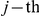 outputs, that is, the function 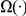 is the averaging function. The mean rule is equivalent to the sum rule (within a normalization factor of 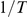), which also appears often in the literature.
  In either case, the ensemble decision is taken as the class 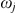, for which the total support 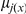 is the largest.
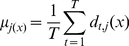
(8)
- **Minimum/Maximum/Median Rule.** As the names imply, these functions simply take the minimum, maximum or the median among the classifiers' individual outputs.
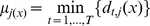
(9)

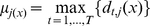
(10)

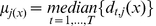
(11)In any of these cases, the ensemble decision is again chosen as the class for which total support is largest. The minimum rule is the most conservative combination rule, as it chooses the class for which the minimum support among the classifiers is largest.
- **Product Rule.** In product rule, supports provided by the classifiers are multiplied. This rule is very sensitive to the most pessimistic classifiers: a low support (close to 0) for a class from any of the classifiers can totally remove the chance of that class to be selected. However, if individual posterior probabilities are estimated correctly at the classifier outputs, then this rule provides the best estimate of the overall posterior probability of the class selected by the ensemble.
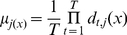
(12)
- **Majority Voting.** Majority voting follows a simple rule: it will vote for the class which is chosen by maximum number of individual classifiers. Let us define the decision of the 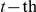 classifier 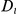 as 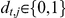, 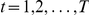 and 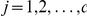 where 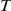 is the number of classifiers and 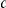 is the number of classes. If the 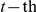 classifier chooses class 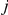, then 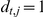, and zero, otherwise. The vote will then result in an ensemble decision for class 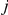 if:
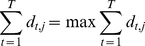
(13)
- **Borda Count.** The Borda count is originally a voting method in which each classifier gives a complete ranking of all possible alternatives. This method, introduced in 1770 by Jean-Charles de Borda, is used if and when the classifiers can rank-order the classes. This can be easily done if the classifiers provide continuous outputs, as the classes can then be rank-ordered with respect to the support they receive from the classifier. However, Borda count does not need the values of these continuous outputs, but just the rankings, hence it qualifies as a combination rule that applies to labels. In standard Borda count, each voter (classifier) rank-orders the candidates (classes). If there are 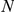 candidates, the first-place candidate receives 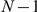 votes; the second-place candidate receives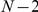, and so on. The candidate ranked last receives zero votes. The votes are added up across all classifiers, and the class with the most votes is chosen as the ensemble decision [32].

Unlike non-trainable combiners, in trainable combiners, a learning process makes the combiner learn to map the base classifiers' outputs to the target space.

- **Decision Template Method.** Decision templates, DTs, were proposed by Kuncheva in [45], for combining continuous valued outputs of an ensemble of classifiers. Decision templates are defined as the average decision profile observed for each class throughout training. Given a test instance 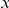, its decision profile is compared to the decision templates of each class, and the class, whose decision template is closest, in some similarity measure, is chosen as the ensemble decision. More specifically, the decision template for 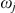, is calculated as
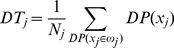
(14)which is the average decision profile obtained from 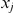, the set (with cardinality 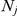) of training instances that belong to true class 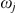. Given an unlabeled test instance 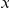, we first construct its 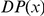 from the ensemble outputs and calculate the similarity 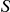 between 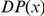 and the decision template 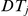 for each class 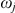 as the degree of support given to class 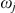.
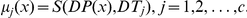
The similarity measure 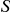 is usually a squared Euclidean distance, obtained as
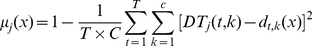
(15)where 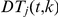 is the support given by the 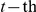 classifier to class 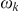 by the decision template 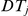. In other words, 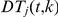 is the support given by the 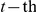 classifier to class 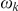, averaged over instances of class 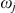. This support should ideally be high when 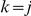, and low otherwise. The second term 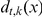 is the support given by the 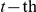 classifier to class 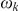 for the given instance 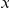. As usual, the class with the highest total support is finally chosen as the ensemble decision.
- **Weighted Averaging Based on Particle Swarm Optimization.** In combining classifiers, since the base classifiers are diverse from each other, it seems that a weighted combination of their outputs yields better results in comparison with simple averaging method (explained in section 3.2.1).
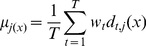
(16)Generally, one popular approach to find the optimal weights is to use evolutionary algorithms like Particle Swarm Optimization [54]. The Particle Swarm Optimization [55] is a stochastic search through the *n*-dimensional space of the real numbers. In Particle Swarm Optimization, each particle in the swarm represents a point in the solution space. The position of a particle is influenced by the best position visited by itself and the position of the best particle in its neighborhood. When the neighborhood of a particle is the entire swarm, the best position in the neighborhood is referred as the global best particle, and the resulting algorithm is referred to as a 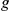-best Particle Swarm Optimization. When smaller neighborhoods are used, the algorithm is generally referred to as a 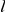-best Particle Swarm Optimization. The performance of each particle is measured using a predefined fitness function, which is related to the problem to be solved. Each particle in the swarm has a current position, 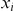, a velocity (rate of position change), 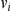, and a personal best position, 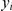. The personal best position of particle 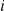 shows the best fitness reached by that particle at a given time. Let 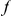 be the objective function to be minimized. Then the personal best position of a particle at time step 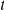 is updated as:
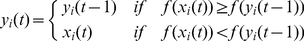
(17)For the 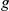-best model, the best particle is determined from the entire swarm by selecting the best personal best position. This position is denoted as 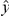. The velocity update equation is stated as:
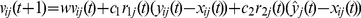
(18)where 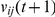 is the velocity updated for the 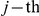 dimension, 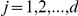. 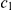 and 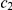 are the acceleration constants, where the former moderates the maximum step size towards the personal best position of the particle and the latter moderates the maximum step size towards the global best position in just one iteration. 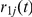 and 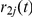 are two random numbers within the range [0,1] and give the Particle Swarm Optimization algorithm a stochastic search property. Velocity updates on each dimension can be clamped with a user defined maximum velocity 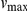, which would prevent them from exploding, thereby causing premature convergence [55].

In Eq. (18), the inertia weight 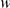 affects the contribution of 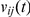 to the new velocity, 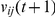. Briefly, this means that if 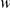 is large, it makes a large step in one iteration (exploring the search space), while if 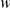 is small, it makes a small step in one iteration, therefore tending to stay in a local region (exploiting the search space). Typically, the inertia weight is set to 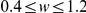. Each particle updates its position using the following equation:
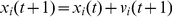
(19)In swarm terminology, particle 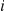 is flying to its new position 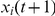. After the new position is calculated for each particle, the iteration counter increases and the new particle positions are evaluated. This process is repeated until some convergence criteria are satisfied.

**Figure 5:**
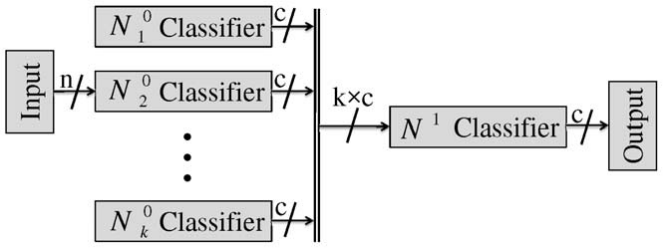
Block diagram of Combined Neural Networks; Stacked Generalization method.

- **Stacked Generalization Method.** Stacked generalization is a technique proposed by Wolpert [46] that extends voting in the sense that the learners (called level-0 generalizers) are not necessarily combined linearly. The combination is made by a combiner system (called level-1 generalizer) that is also trainable. The general framework of this method (see Figure 5) consists of two levels. The first level, level-0, is formed by base classifiers which are trained using the input data and the target output. The output of level-0 is then used as the input of level-1. As is shown in Figure 5, a set of 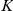 “level-0” networks from 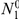 to 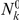 are arranged as the first layer, and their outputs are combined using a “level-1” network 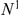.

#### Proposed Method: Modified Stacked Generalization Method

In this section, we introduce our proposed method, the Modified Stacked Generalization, and after justifying its use, we will demonstrate how this method improves the performance of a classification problem.

**Figure 6:**
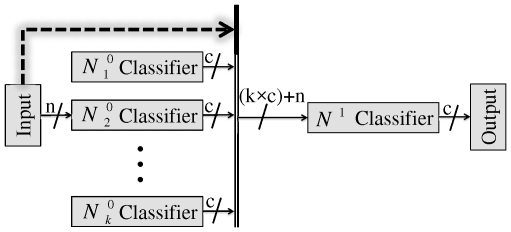
Block diagram of Combined Neural Networks; Modified Stacked Generalization method.

In combination methods discussed in sections 3.1 and 3.2, the outputs of the diverse base classifiers are combined together in different ways. In section 3.1 a fix rule combines the classifiers' results, independent from knowing how the problem space is broken. In section 3.2 we described the Stacked Generalization method in which, during the training phase, the combiner learns proper weights for combining the outputs of the base classifiers for an input sample. These weights are then used in the test phase to optimally combine the outputs of the base classifiers for each test sample. In this method, as the weights for combining the outputs of the base classifiers are obtained during a learning process which endows the power of generalization to the model, we expect the model to have a better performance in comparison with the fix combination rules. However, since the learning process of the combiner is only depended on the outputs of the base classifiers, it does have no information about the relation between the input problem space and the votes of the base classifiers. As the result, the combiner just learns the mapping between the outputs of the base classifiers and the target classes of the input samples, rather than learning the mapping between the input samples and their corresponding target classes. In other words, the combiner never sees the distribution of the input problem space, and therefore it cannot assign the *optimal weights* for combining the outputs of the base classifiers regarding the input sample [48] [56]. Unlike the Stacked Generalization method, in this paper we propose to feed the combiner with both the input sample and outputs of the base classifiers simultaneously (figure 6). One important advantage of this change is that the combiner obtains an understanding of the relation between the input problem space and the votes of the base classifiers. In other words, for each input sample, the combiner gives the optimal weights for aggregating the outputs of the base classifiers regarding the position of the input sample in the problem space and also its knowledge about the area of expertise of the base classifiers.

In the training phase, by looking at the input sample and the base classifiers' outputs, the combiner learns how to divide the problem space between the base classifiers. In the testing phase and for each input sample, the combiner uses the representation of the input sample and the previous knowledge about the expertise of the base classifiers to optimally determine the weights to aggregate the votes of base classifier(s).

## Results

In this section, after a description of preprocessing module and feature extraction, as we are proposing a new combining strategy, we continue with important issues for choosing the base classifiers' parameters. We then show the improvement of the proposed method over some combining methods followed by error analysis using some evaluation metrics to properly assess the effectiveness of our proposed algorithm. We finally bring the comparison with other related methods in the literature.

### Preprocessing and Feature Extraction

**Figure 7:**
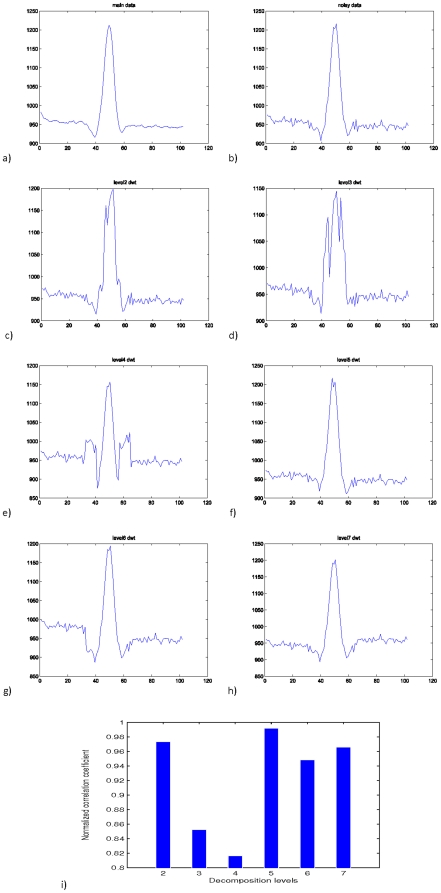
Results of wavelet denoising. a) original signal, b) noisy signal, c–h) results of denoising procedure obtained by using wavelets: db1 with decomposition levels (2–7), i) Comparative plot of correlation coefficients with selected decomposition levels of wavelet filter for signal under denoising.

For denoising we have used the Daubechies wavelet functions (db1) with decomposition level of five. This selection is based on our extensive experiments among which one sample is shown in (Figure 7). In this figure, a sample noisy signal (the noise is normally distributed with zero mean and variance) is shown along with the results of denoising procedure obtained by using wavelet db1 with decomposition levels 2–7 (Figure 7b–7h). This procedure is based on decreasing the noise content in high frequency components (decomposition coefficient) of signal which is performed using the soft-thresholding method described in [20]. We have compared correlation coefficients of six decomposition levels of denoised signal in Figure 7i.

In this study, we have used the Savitsky–Golay filtering method for smoothing of the ECG signals [20]. The filter coefficients are achieved by the un-weighted linear least-squares fitting method using a polynomial function. Because of this, the Savitsky–Golay filter is also called a digital smoothing polynomial filter or a least-squares smoothing filter. A higher degree of polynomial makes it possible to achieve high level smoothing without attenuation of the data features. The Savitsky–Golay filtering method is often used for frequency information or spectroscopic (peak) information. For the first type, it conserves the high-frequency components of the signal and for the second type it conserves higher moments of the peak. In the feature extraction stage a combination of morphological and timing features are used. These features describe the basic shape of the signals and position of waves within a given window of beats. The extracted parameters that describe the basic shape of the beats are: amplitude of P-peak (ampP), amplitude of Q-valley (ampQ), amplitude of R-peak (ampR), amplitude of S-valley (ampS) and amplitude of T-peak (ampT). Features that describe wave position within a beat window are: position of P-peak (posP), position of Q-valley (posQ), position of R-peak (posR), position of S-valley (posS) and position of T-peak (posT). The time duration between PVC beats contains useful information about their types. So we use a feature called rat RR, which is defined as the time ratio between the last beat to the next one.

Thus, ten ECG morphological features are extracted, as well as one timing interval feature. To extract this feature we propose a two-steps method. The first step involves the cutting of the normal and PVC and other beats by making use of the annotation files which exist in MIT–BIH arrhythmia database. The second step involves identification of peaks and valleys in every normal or abnormal beat and obtaining their respective amplitudes and positions. In order to break to normal and abnormal beats, we process annotated file records from MIT–BIH database. For example to extract normal beats, the algorithm examines the annotation file which contains the sample number of the normal beat. Then it creates a matrix with rows equal to the number of normal beats. An R-wave detector is required to initialize our computer-aided ECG classification process. Next, the algorithm saves 40 samples surrounding the target normal beat from all the recorded samples. The sample beat itself is also saved in the final matrix. We extracted the abnormal beats in the same manner too. After classification of normal and abnormal beats, peaks and valleys are detected. For this purpose, we implemented the Al-Alaoui algorithm [57]. The peak and valley detector correctly detects the P, Q, R, S, and T waves. Sometimes, especially in the case of arrhythmia, it is possible for the algorithm to recognize extra peaks or valleys. Since the P and the T waves exist at the beginning and the end of each window, respectively, in such a case the first peak is set as the P and the last peak as the T wave; other peaks are hence rejected. Similarly, the algorithm marks the nearest valley at the left of center of the beat as the Q, and the nearest valley to the right of center of the beat as the S wave. We extracted ten ECG morphological features, as well as one timing interval feature.

### Base classifiers structure selection

An important issue in combining classifiers is the diversity of the classifiers in learning the input data. When the base classifiers of a combining structure are diverse (i.e., they learn different areas of the input space), they become specialized in specific areas of the input space, and consequently have fewer errors in those areas. Thus, combining the outputs of classifiers that are perfectly diverse, improves the overall performance. For diversifying the base classifiers, different training parameters and classifiers with different structures have used.

**Figure 8:**
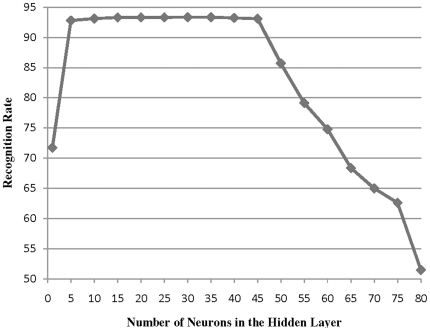
Recognition rate of an MLPs neural network with different number of neurons in the hidden layer.

In our combining methods we have used MLPs classifiers with Back Propagation training algorithm as our base classifiers. For each classifier, some parameters are pre-specified according to the characteristics of our classification task, e.g. the number of input nodes is equal to the number of extracted features of each input sample, while the number of output nodes is specified on the basis of the number of classes. Some parameters including the initial weights and the learning rate are specified by trial and errors. The number of neurons in the hidden layer of the MLPs is also determined via trial and error method. In this manner, as graphically depicted by figure 8, we have increased the number of hidden neurons from 5 to 80 in order to find the optimal domain of the number of hidden neurons. Eventually, the number of epochs is determined with cross validation method. Table 1 illustrates the described parameters for three diverse base classifiers.

**Table 1:** Recognition rates and other parameters of the base classifiers for combining methods using three base classifiers.

Base Classifier	Number of Neurons in the Hidden Layer	Number of Epochs	Initial Weights Range	Recognition Rates (%)
Classifier 1	17	1700	[−2 2]	93.71
Classifier 2	22	2700	[−3 3]	93.77
Classifier 3	25	2000	[−4 4]	93.78

### Application of Different Combining Methodologies and Proposed Method to ECG Signals

For evaluation, we used the same diverse classifiers as the base classifiers and then their outputs are aggregated via different combining strategies. Table 2 shows the results of this evaluation and Table 3 indicates the standard deviations as well as the number of neurons in the hidden layer of the best topologies for Stacked Generalization and Modified Stacked Generalization.

**Table 2:** Recognition rates for different combining methods as well as the proposed method with different number of experts.

Number of Experts	2	3	4	5
Method				
Maximum Rule	94.04	93.30	91.03	90.32
Minimum Rule	94	94.09	93.71	93.31
Average Rule	94.01	94.05	94.19	94.20
Product Rule	93.80	93.63	92.12	93.40
Majority Voting	93.82	93.93	93.94	93.12
Borda Count	93.80	94.09	93.94	93.70
Decision Templates	93.21	93.43	93.38	93.47
Weighted Averaging Based on Particle Swarm Optimization	94.26	94.26	94.33	94.41
Stacked Generalization	94.52	94.7	94.49	94.51
Modified Stacked Generalization	94.8	95.2	94.53	94.62

**Table 3:** Standard deviation and number of neurons in the hidden layer of the best topologies for Stacked Generalization method and Modified Stacked Generalization method.

Number of Experts	2	3	4	5
Standard Deviation				
Stacked Generalization method	0.50	0.40	0.54	0.70
Modified Stacked Generalization method	0.40	0.35	0.50	0.56
Number of Hidden Neurons of the Best Topology				
Stacked Generalization method	30	30	15	10
Modified Stacked Generalization method	40	45	35	30

**Figure 9:**
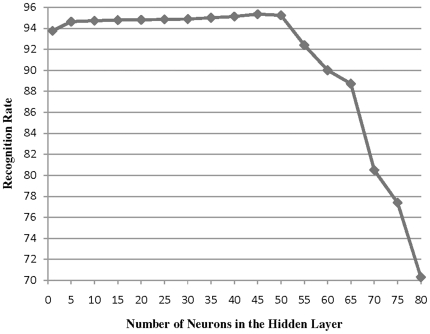
Recognition rate of the combiner in the Modified Stacked Generalization method with different number of neurons in the hidden layer.

In all experiments, a number of base classifiers are first created. In test phase, for Max, Min, Average and Product methods, their outputs are combined with corresponding rules. In Stacked Generalization scheme, the outputs are used as inputs to second level classifier to learn the mapping between the base classifiers' outputs and the target of test sample. In MSG method, the outputs and a direct input pattern are used as inputs to a second level meta-classifier to learn the mapping between the base classifiers' outputs and the actual correct labels. The input of the combiner (second-level classifier) in conventional Stack Generalization has nine elements. In our proposed method, in addition to these nine inputs, the combiner also receives the original input pattern (which has eleven elements). Altogether, the input of the combiner would be a 20-element vector. In Table 2, the recognition rates of different combining methods are listed. As shown in figure 9 to find the best topology of the combiner, we employed the same strategy as for the base classifiers by increasing the number of hidden neurons from 5 to 80 and investigating the best recognition rate on our validation set. The optimal number of hidden neurons was found to be 45 for the Modified Stack Generalization method.

Here, two relatively important points can be derived from Table 2. The first one is that both of the trainable combining methods (Stacked Generalization and Modified Stacked Generalization) are of higher performance in comparison with the non-trainable combining methods. Secondly, regardless of the number of experts used in the combining structure, the proposed combining method has the best performance and the least variance.

### Error Analysis

We analyzed the performance of our proposed method based on some evaluation metrics described in [56] to properly assess the effectiveness of the method. Classification results of the classifiers were displayed by a confusion matrix. A confusion matrix is square matrix that contains information about actual and predicted classifications done by a classification system. The confusion matrices showing the classification results of the base classifiers as well as the confusion matrix of the Modified Stacked Generalization method are given in Table 4. From these matrices one can tell the frequency with which an ECG beat is misclassified as another.

**Table 4:** Confusion matrix of the base classifier 2, for the 3 class ECG signal classification.

Classifier	Output Result	Desired Result		
		Normal Beat	PVC Beat	Other Beats
Base Classifier 1	Normal Beat	7888	15	537
	PVC Beat	163	390	158
	Other Beats	139	22	5654
Base Classifier 2	Normal Beat	7944	4	516
	PVC Beat	106	413	178
	Other Beats	140	10	5655
Base Classifier 3	Normal Beat	7966	5	554
	PVC Beat	135	408	169
	Other Beats	89	14	5626
Combiner	Normal Beat	7969	3	336
	PVC Beat	111	413	137
	Other Beats	110	11	5876

The produced ECG signal classes are in table rows while the table columns are the classes of the reference ECG signal.

The test performance of the classifiers can be determined by the computation of the following four statistical parameters:

1. Specificity: number of correctly classified normal beats over total number of normal beats.
2. Sensitivity (PVC): number of correctly classified premature ventricular contraction beats over total number of premature ventricular contraction beats.
3. Sensitivity (other): number of correctly classified other beats over total number of other beats.
4. Overall classification accuracy: number of correctly classified beats over number of total beats.

These parameters are computed as shown in Table 5.

**Table 5:** The values of statistical parameters.

Statistical Parameter	Percentage
Specificity	97.3
Sensitivity (PVC)	96.72
Sensitivity (Others)	92.55
Overall Classification Accuracy	95.26

The last part of this section is the comparison of the recognition rates for the proposed method with some popular classifiers in the literature (see table 6).

**Table 6:** Comparison of the recognition rates of the proposed method with some popular classifiers in the literature.

Classification Scheme	Recognition Rate
Radial Basis Function Neural Network	89.11
Support Vector Machine Classifier	93.43
Multilayer Perceptrons (MLPs) with Back Propagation Algorithm [21]	93.26
Stacked Generalization with Back Propagation Algorithm	94.8
Modified Stacked Generalization with Back Propagation Algorithm	95.2

In summary, this paper presented a new combining method for classification of the ECG beats based on Stacked Generalization. By aggregating the original input patterns to the outputs of the base classifiers, and as the result, by increasing the knowledge of the combiner, we helped it make a better decision according to the base classifiers' decisions. Experimental results and higher recognition rates of the proposed method support our claim that such additional knowledge lets the combiner to find a better solution.
